# The Different Substrate Characteristics of Arrhythmogenic Triggers in Idiopathic Right Ventricular Outflow Tract Tachycardia and Arrhythmogenic Right Ventricular Dysplasia: New Insight from Noncontact Mapping

**DOI:** 10.1371/journal.pone.0140167

**Published:** 2015-10-21

**Authors:** Van Buu Dan Do, Wen-Chin Tsai, Yenn-Jiang Lin, Satoshi Higa, Nobumori Yagi, Shih-Lin Chang, Li-Wei Lo, Fa-Po Chung, Jo-Nan Liao, Yen-Chang Huang, Chao-Shun Chan, Hung-Kai Huang, Yu-Feng Hu, Hsuan-Ming Tsao, Shih-Ann Chen

**Affiliations:** 1 Division of Cardiology, Taipei Veterans General Hospital, Taipei, Taiwan; 2 Division of Cardiology, Department of Medicine, Hualien Tzu-Chi General Hospital, Taipei, Taiwan; 3 Faculty of Medicine, Institute of Clinical Medicine, and Cardiovascular Research Institute, National Yang-Ming University, Taipei, Taiwan; 4 Cardiac Electrophysiology and Pacing Laboratory, Division of Cardiovascular Medicine, Makiminato Central Hospital, Okinawa, Japan; 5 Division of Cardiovascular Medicine, Nakagami Hospital, Okinawa, Japan; 6 Cardiology, National Yang Ming University Hospital, I-Lan, Taiwan; Loyola University Chicago, UNITED STATES

## Abstract

**Background:**

The aim of this study was to investigate the different substrate characteristics of repetitive premature ventricular complexed (PVC) trigger sites by the non-contact mapping (NCM).

**Methods:**

Thirty-five consecutive patients, including 14 with arrhythmogenic right ventricular cardiomyopathy/dysplasia (ARVC) and 21 with idiopathic right ventricular outflow tract tachycardia (RVOT VT), were enrolled for electrophysiological study and catheter ablation guided by the NCM. Substrate and electrogram (Eg) characteristics of the earliest activation (EA) and breakout (BO) sites of PVCs were investigated, and these were confirmed by successful PVC elimination.

**Results:**

Overall 35 dominant focal PVCs were identified. PVCs arose from the focal origins with preferential conduction, breakout, and spread to the whole right ventricle. The conduction time and distance from EA to BO site were both longer in the ARVC than the RVOT group. The conduction velocity was similar between the 2 groups. The negative deflection of local unipolar Eg at the EA site (EA slope_3,5,10ms_ values) was steeper in the RVOT, compared to ARVC patients. The PVCs of ARVC occurred in the diseased substrate in the ARVC patients. More radiofrequency applications were required to eliminate the triggers in ARVC patients.

**Conclusions/Interpretation:**

The substrate characteristics of PVC trigger may help to differentiate between idiopathic RVOT VT and ARVC. The slowing and slurred QS unipolar electrograms and longer distance from EA to BO in RVOT endocardium suggest that the triggers of ARVC may originate from mid- or sub-epicardial myocardium. More extensive ablation to the trigger site was required in order to create deeper lesions for a successful outcome.

## Introduction

Focal ventricular tachycardia (VT) could be observed in both idiopathic right ventricular outflow tract (RVOT) tachycardia and arrhythmogenic right ventricular cardiomyopathy/dysplasia (ARVC) [1,2]. ARVC is an inherited cardiomyopathy characterized by progressive fibro-fatty replacement of the right ventricular (RV) myocardium predisposing to ventricular tachycardia and death [3]. Right ventricular outflow tract tachycardia is a benign condition, traditionally considered to be a primary electrical disease in the absence of structural heart disease [1,4]. Radiofrequency (RF) ablation is considered the first-line treatment of symptomatic RVOT in patients with structurally normal heart while its role in ARVC is more limited [5,6]. Recent study showed a high prevalence of focal VTs in patient with ARVC and an ablation strategy targeting the focal triggers of these VT has proven to be effective [7]. However, conventional mapping and ablation of VT can be technically challenging, requiring significant fluoroscopic and procedural times. A noncontact mapping (NCM) system incorporating a multielectrode array (MEA) has been demonstrated to facilitate the identification of ectopic focus, because it is able to reconstruct the precise geometry in the atrium and ventricle and localize the trigger just in one beat [8–11]. In addition, it also helps to characterize the abnormal substrate and predicts the successful and potentially difficult ablation sites [9–11]. The aim of this study was to investigate the different substrate characteristics of trigger sites in idiopathic RVOT VT and ARVC by the NCM method.

## Methods

### Study population

Between October 2008 and July 2012 a total of 35 consecutive patients with RVOT VT which was refractory to at least one antiarrhythmic drug, in the absence of coronary disease, surgical scars or left ventricular dysfunction were enrolled for electrophysiological study (EPS) and catheter ablation guided by the NCM. Among 35 patients, 21 were diagnosed with idiopathic RVOT VT and 14 were diagnosed with ARVC on the basis of the modified Task Force criteria for diagnosis of ARVC proposed by an international working group [12]. The VT trigger was identified as the first beat of the inducible VT or the single PVC if no VT was inducible during the procedure. In case of ARVC, the first beat of the inducible VT which has the same morphology with the clinical RVOT VT was considered as the dominant trigger. As a result, only 1 dominant trigger for each patient was included in this study. The RVOT VT origin was determined by electrocardiographic criteria and confirmed in all cases at EPS.

### Electrophysiological Study and programmed ventricular stimulation

This study was conducted at the Taipei Veterans General Hospital in Taiwan, approved by the institutional review board of the Taipei Veterans General Hospital (IRB: 2011-05-003IC) and Department of Health, Taiwan; and at the Makiminato Central Hospital, Okinawa, Japan, approved by the institutional review board of Makiminato Central Hospital, Okinawa, Japan (IRB: MCH-26-1). After written consent was obtained, EPS was performed in the fasting and non-sedated state. Before the study, all antiarrhythmic drugs except amiodarone were discontinued for at least 5 half-lives. If there was no VT at baseline, the programmed ventricular stimulation protocol with or without Isoproterenol infusion (1–4 μg/min) was performed as previously described [5]. If an induced VT was hemodynamically tolerated, pacing from the mapping catheter at a cycle-length 20-30ms faster than the tachycardia cycle-length was performed, observing the entrainment response. A site was considered a VT mid-isthmus only if it demonstrated (1) concealed fusion on all 12 ECG leads during entrainment, (2) the post-pacing interval was within 30ms of the VT cycle-length, (3) the stimulus-electrogram interval was within 30ms of the electrogram-QRS interval following entrainment and 4) the local electrogram to QRS interval was between 30 and 70% of the VT cycle-length. Confirmed VT isthmus sites were annotated on the map after VT termination in sinus rhythm.

### Noncontact mapping of focal VT

The use of NCM system in our laboratory has been previously described in detail [10,11]. In brief, the system consisted of a catheter (9-F) with a multi-electrode array (MEA) surrounding a 7.5-ml balloon mounted at the distal end. Raw data detected by the MEA was amplified and digitally transferred to a computer workstation.

The MEA catheter was deployed in the RVOT over a 0.035-inch guidewire, which was advanced 5 cm into the pulmonary artery. The system located the 3-dimensional (3D) position of the electrodes on any desired catheter relative to the MEA using a navigation signal. Navigation provided the means to define a model of the chamber anatomy and to track the position of standard contact catheters within the chamber relative to labeled points of interest, such as anatomic structures or critical zones of conduction. Simultaneous virtual unipolar electrograms (VUE) were mathematically reconstructed and displayed on the anatomic model, producing isopotential or isochronal color maps. Signals for both electrograms were filtered with a bandwidth of 2 to 300 Hz. Virtual unipolar electrograms could also be selected and displayed from any site of interest on the anatomic model using the mouse pointer.

### Validation of Noncontact Electrogram

The NCM electrogram was validated as described in our previous studies [10,13]. Briefly, the EnGuide navigation signals were simultaneously recorded from each site for geometric annotation of location and for generation of virtual electrograms that can be compared with the associated contact electrograms. The sites were randomly chosen and approximately evenly spaced. Both the noncontact mapping system and the conventional mapping system were set up for simultaneous recording of both contact and noncontact electrograms. Signals for both contact and noncontact electrograms were filtered with a bandwidth of 2–300 Hz. Electrogram morphologies, activation time difference and electrical voltage between contact and noncontact electrograms that were taken from the same endocardial sites were compared by use of a well-described template comparison algorithm [14,15].

### Ventricular substrate analysis

**Fig 1:**
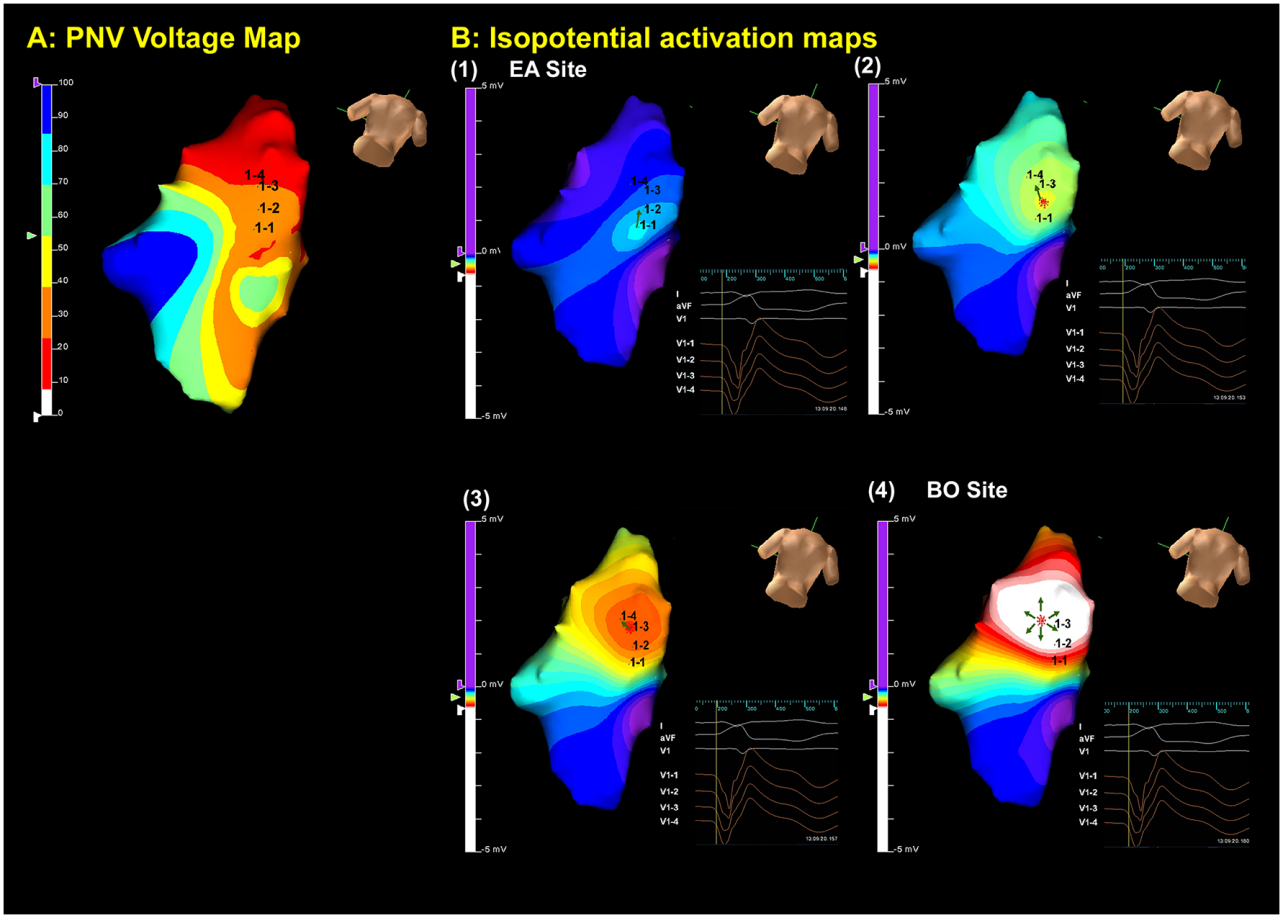
Dynamic substrate map and isopotential maps of noncontact mapping. (A) Normalized peak negative voltage (PNV) distribution of the RV in a posterior caudal view. The orange border zone rerepresents areas with voltages around 30% of the peak negative potential. (B) Isopotential map shows the activation sequence (frames 1–4). Color scale has been set so that white indicates the most negative potential and purple indicates the least negative potential. Virtual electrodes (V1-1 to V1-4) are placed along the propagation of activation wavefront from EA site (Frame 1) to BO site (Frame 4). The green arrows indicate the activation wavefron propagating from EA to BO site, then spreading out at BO site. The virtual unipolar electrograms reveal a QS pattern at the origin.

**Fig 2:**
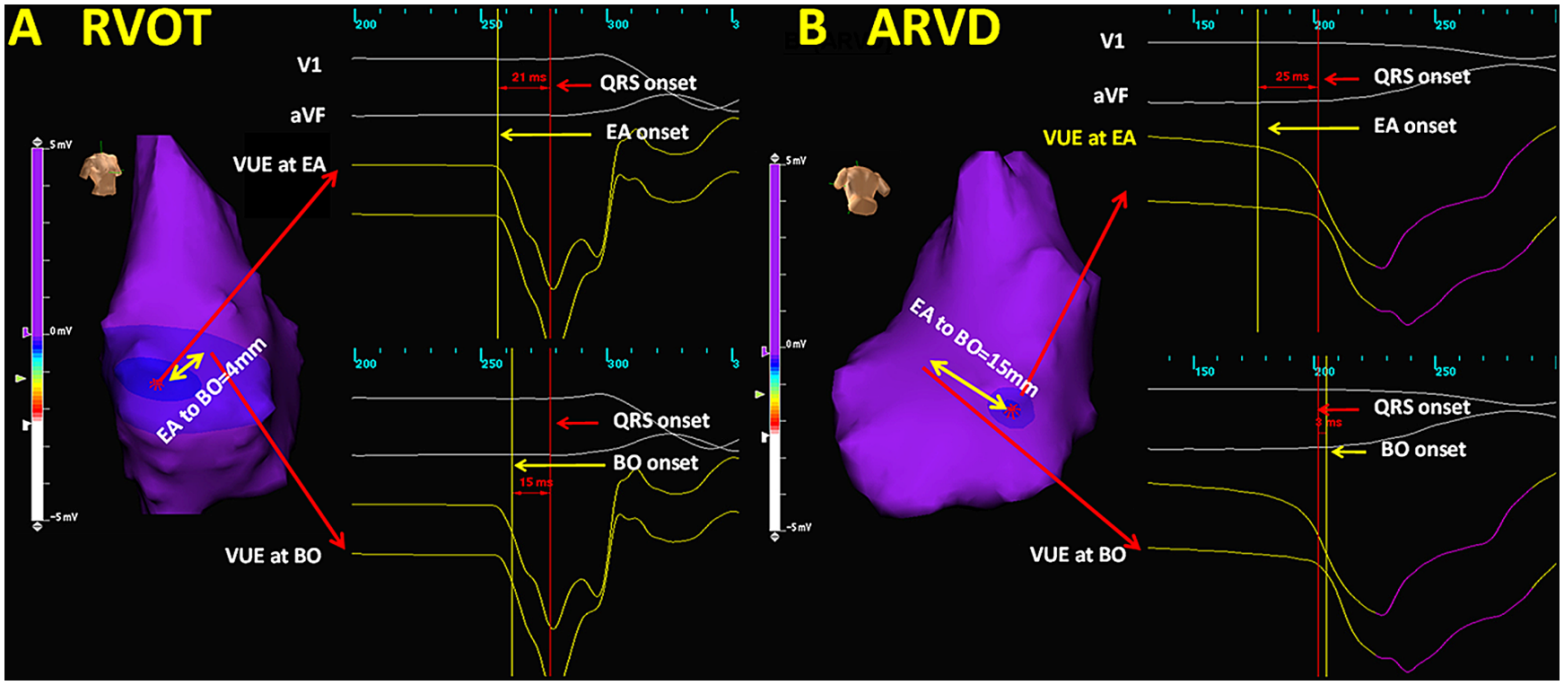
Representative isopotential maps and intracardiac electrogram of RVOT and ARVC triggers. The yelow and red line indicate the onset of local VUE and QRS, respectively. As for RVOT trigger, the VUE at the EA precedes the QRS onset (EA to QRS interval = 21ms) and the VUE at BO precedes the QRS onset (BO to QRS interval = 15ms). As for ARVC trigger, the VUE at the EA precedes the QRS onset (EA to QRS interval = 25ms) and the VUE at BO follows the QRS (BO to QRS interval = -3ms).

On each NCM activation map, EA and BO sites were marked (Fig 1). The EA was defined as the earliest site showing a single spot of isopotential map and a QS pattern of noncontact VUE. The BO site was the site identified by the white spot within the red zone where rapid centrifugal electrical propagation originated from and local VUE showed a sudden increase of peak negative potential after VT depolarized. The quantification of VUE was represented in Fig 2. The EA to QRS interval in millisecond (ms) was the interval between the onset of VUE at EA site (VUE_EA_) to QRS onset. The BO to QRS interval (ms) was the interval between onset of VUE at BO site (VUE_BO_) to QRS onset. The temporal and spatial difference between EA and BO site was measured by conduction time (ms) and distance (mm), respectively. The conduction velocity from EA to BO sites was computed by dividing distance between EA and BO by the conduction time. The EA slope value was the amplitude in milivoltage (mV) of the negative deflection of VUE_EA_ measured at 3ms, 5ms and 10ms after the Eg onset. The BO slope value was the amplitude of the negative deflection of VUE_BO_ measured at 3ms, 5ms and 10ms after the Eg onset. All the measurements were made by physicians who were blinded to the diagnosis of VT.

### High-density voltage mapping

A dynamic substrate map (DSM) was also created during sinus rhythm to help determine the substrate characteristics of the triggers. The mean peak negative voltage (PNV) of the global RV was analyzed from the negative portion of the unipolar electrograms, which were obtained simultaneously from 256 virtual mapping sites equally distributed throughout the RV. The normalized PNV (i.e., the relative ratio to the maximal PNV of one selected beat) in each virtual electrogram was used to produce the DSM of the entire chamber. Low voltage zones (LVZ) was an area with normalized PNV of less than 37%, which predicted slow conduction in the right atrium as demonstrated in our previous observation [11].

### Catheter ablation and Follow-up

Catheter ablation (40 to 50W, 50°C to 60°C, 40 seconds) was performed using a 4-mm electrode-tipped ablation catheter connected to an EPT-1000 generator (Boston Scientific Co). We first delivered RF energy at the EA sites. If initial ablation was unsuccessful at the EA site, then the RF ablation target was switched to the BO site. The pace mapping during sinus rhythm and entrainment mapping during VT were also used to confirm an appropriate site for ablation. The ablation procedure was considered successful if the VT was eliminated during the ablation and/or became non-inducible with programmed ventricular stimulation with or without Isoproterenol infusion. After hospital discharge, the patients were followed up closely (every 1 to 3 months) in the outpatient clinic. Long-term efficacy was assessed clinically on the basis of the resting surface ECG, 24-hour Holter monitoring, and clinical symptoms. The recurrence was defined as recurrence of sustained VT, nonsustained VT, or greater than 1000 ventricular PVCs [16], as confirmed by morphology criteria using 24-hour Holter monitoring.

### Statistical Methods

All continuous data were expressed as the mean ± standard deviation or median (interquartile range) when appropriate. Categorical data were presented as percentage. Differences between the continuous values were assessed using the Student T test for normally distributed continuous variables or the Mann-Whitney U test for skewed variables. A chi-square test with Yates' correction or Fisher's exact test was used for categorical data. The optimal cutoff values for identifying the ARVC group were generated from receiver-operating characteristic (ROC) curves. The EA to BO distance and EA slope value at 5ms (EA slope_5_) was used for determining the cutoff value according to the greater area under the ROC curve (AUC: EA to BO distance 0.768, EA slope_5_ 0.745). A value of P < 0.05 was considered to be statistically significant. All statistical analyses were performed using commercial statistical SPSS version 20.0 software (SPSS, Chicago, IL, USA).

## Results

### Patient Characteristics

The patient population consisted of 35 patients, including 14 (40%) with ARVC and 21 (60%) with idiopathic RVOT VT. The baseline clinical characteristics of patient were presented in Table 1. Patients were of similar age in the two groups. There was a predominance of male patients in the ARVC group (57.1% vs. 19%, p = 0.031). There was no significant difference in family history between patients with ARVC and RVOT tachycardia. Three of 14 (21.4%) patients in ARVC group had ICD implantation before ablation. More patients in ARVC group presented with syncope or near syncope than in RVOT group, although this difference did not reach statistical significance (50% vs. 28.6%, p = 0.35). The amount of premature ventricular contraction (PVC) per day and the VT induced by programmed ventricular stimulation before ablation did not differ between the 2 groups. Both groups had similar left ventricular ejection fraction (LVEF), but ARVC patients had a lower right ventricular ejection fraction (RVEF, 44±12 vs. 57±5, p = 0.001). ARVC patients had a median Task Force Score of 3, ranging from 2–7, which was higher than those of RVOT-VT patients.

**Table 1:** Baseline Clinical Characteristics of 35 Patients.

Variables	ARVC (n = 14)	RVOT (n = 21)	P value
Age (years)	43.8±11	43.7±14.5	0.967
Male (%)	57.1	19	0.03
Family history (%)	7.1	4.8	1.00
ICD implant (%)	21.4	0	0.056
Syncope/Near syncope (%)	50	28.6	0.288
PVC amount	12753±11165	15343±9345	0.579
Inducible VT (%)	50	57.1	0.739
LVEF*	56±7%	61±3%	0.062
RVEF*	44±12%	57±5%	0.001
Task Force score ^†^	3 (2–7)	1 (0–1)	<0.001
Number of major criteria	1 (0–3)	0 (0–0)	<0.001
Number or minor criteria	2 (1–3)	1 (0–1)	<0.001

* Measured by ventriculogram

^†^ Data are presented as median (range).

ARVC = arrhythmogenic right ventricular cardiomyopathy; ICD = implantable cardioverter-defibrillator; LVEF = left ventricular ejection fraction; NS = nonsignificant; PVC = premature ventricular contraction; RVEF = right ventricular ejection fraction; RVOT = right ventricular outflow tract; VT = ventricular tachycardia.

### Characteristics of the substrate at the triggers

Overall 35 focal triggers were selected (14 in ARVC group and 21 in ROVT group; EA and BO sites were identified on the NCM for all patients. NCM clearly showed the anatomic locations and activation wavefronts from origins with preferential conduction which activated the myocardium surrounding the origin (Fig 1). The NCM findings were presented in Table 2. There was no significant difference in the EA to QRS interval between the ARVC and the RVOT groups (22.8±6.3mm vs. 22.4±6.8mm, P = 0.85). The BO to QRS interval was shorter in ARVC patients than in RVOT patients (4.8±8.3ms vs. 12.9±8.2ms, P = 0.04). Consequently, the conduction time from EA to BO site was significantly longer in the ARVC than in the RVOT group (18.1±11.7ms vs. 9.5±5.5ms, P = 0.03). The conduction velocity was similar between the 2 groups (1.1±0.8 m/s vs. 1.0 ±0.7 m/s, P = 0.64), which, in turn, resulted in a longer distance from EA site to BO site in ARVC group than in RVOT group (14.9±9.5mm vs. 7.6±4mm, P = 0.02).

**Table 2:** Noncontact mapping findings of triggers.

Variables	ARVC (n = 14)	RVOT (n = 21)	P value
EA to QRS interval (ms)	22.8±6.3	22.4±6.8	0.85
BO to QRS interval (ms)	4.8±8.3	12.9±8.2	0.04
**EA to BO site**			
Conduction time (ms)	18.1±11.7	9.5±5.5	0.03
Distance (mm)	14.9±9.5	7.6±4	0.02
Conduction velocity (m/s)	1.1±0.8	1±0.7	0.64
**EA Unipolar Eg slope value (mV)**			
3 ms	0.69±0.47	1.55±1.14	<0.01
5 ms	1.06±0.70	2.6±2.04	<0.01
10ms	3.01±2.59	5.64±4.11	0.03
**BO Unipolar Eg slope value (mV)**			
3 ms	4.14±2.10	5.40±2.87	0.17
5 ms	4.93±2.68	6.54±3.25	0.13
10ms	6.97±4.45	8.16±3.89	0.41
Mean global PNV of RV (mV)	3.9±2.8	4.8±2.9	0.34
**Normalized PNV (%) of the maximal voltage**			
EA site	25±16	42±29	0.04
BO site	28±17	45±28	0.03

BO = breakout; EA = earliest activation; Eg = electrogram; PNV = peak negative value; Other abbreviations are the same as Table 1.

The unipolar Eg slope value at the EA site was significantly greater at 3 subsequent timings (3, 5, 10ms) in the RVOT group than in ARVC group (1.55±1.14 vs. 0.69±0.47 mV, P<0.01; 2.6±2.04 vs. 1.06±0.7 mV, P<0.01; and 5.64±4.11 vs. 3.01±2.59 mV, P = 0.03, respectively), which implied that the negative deflection of local unipolar Eg at the EA site was steeper for those in the RVOT, as compared to ARVC patients. However, the unipolar Eg slope values of the BO site at 3 ms, 5 ms, and 10 ms was not significantly different between ARVC and RVOT patients (4.14±2.1 vs. 5.4±2.87 mV; 4.93±2.68 vs. 6.54±325 mV; 6.97±4.45 vs. 8.16±3.89 mV, respectively, P = nonsignificant [NS] for all).

A combination of cutoff values for EA to BO distance of >6mm and EA slope_5_ of ≤2.2mV could differentiate the ARVC group from RVOT group, as manifested by a sensitivity of 79% and specificity of 76% (AUC 0.88).

### Characteristics of the substrate at the global substrate

The mean global PNV of unipolar electrograms in the RV endocardium was similar between RVOT and ARVC patients (3.9±2.8mV vs. 4.8±2.9mV, P = 0.34). The normalized unipolar PNV at the EA and BO site was lower in ARVC group than in RVOT group (25±16% vs. 42±29%, P = 0.04 and 28±17% vs. 45±28%, P = 0.03, respectively), indicating that the triggers occurred in the LVZs in the ARVC patients. Three isthmuses were identified in ARVC patients during entrainment. All central isthmus and exit site were located in low voltage area and normal voltage area respectively. Late potential were identified in one central isthmus (33.3%).

### Catheter ablation and follow-up

The results of catheter ablation were shown in Table 3. The incidence of triggers in the RVOT free wall was more prevalent in the ARVC patients compared with RVOT VT (35.7% vs. 9.5%, P = 0.09) whereas the triggers from RVOT septum was more frequent in the RVOT group (90.5% vs. 64.3%, P = 0.09).

**Table 3:** Radiofrequency ablation and follow-up.

Variables	ARVC (n = 14)	RVOT (n = 21)	P value
**Location of trigger within RVOT (identified by Ensite)**			
Septum	9 (64.3%)	19 (90.5%)	0.09
Free wall	5 (35.7%)	2 (9.5%)	0.09
RF ablation number for trigger	18.1±7.5	8.9±3.7	<0.01
**Follow-up**			
Recurrence	6/14 (42.9%)	10/21 (47.6%)	0.78
Time to recurrence (months)	17.7±6	4.2±2	<0.01
Substrate modification during 2^nd^ procedure	5/14 (35.7%)	0 (0%)	<0.01
Epicardial ablation during 2^nd^ procedure	3/14 (21.4%)	0 (0%)	0.06

RF = radiofrequency; Other abbreviations are the same as Table 1.

Regarding RF ablation, more RF applications were required to eliminate the triggers in ARVC patients (18.1±7.5 pulses vs. 8.9±3.7 pulses, P<0.01). During a mean follow-up period of 11.3±10.6 months, 16 (45.7%) patients (6 of ARVD and 10 of RVOT groups) developed recurrences. Patient of ARVC group developed recurrence later than RVOT group (17.7±6 months vs. 4.2±2 months, P<0.01). Of the 6 patients who had recurrence in ARVC group, 1 developed non-sustained VT and 5 had recurrent PVCs with morphology similar to the one of index procedure. Except 1 patient refused a second procedure, 5 of 14 (35.7%) patients with ARVC underwent substrate ablation in the second procedure. Among these 5 patients, 3 underwent epicardial mapping and ablation after failed endocardial approach. In these 3 patients, abnormal epicardial substrate was observed in the corresponding endocardial sites as breakout of the triggers. Recurrence of endocardial PVCs at the same endocardial sites was observed. Of 10 patients who had recurrence in RVOT group, 3 developed non-sustained VT and 7 had recurrent PVCs. Among these 10 patients, 4 had different morphology of PVCs compared with the original one. No patients in the RVOT group underwent a second procedure.

## Discussion

### Main Findings

The present study, to the best of our knowledge, is the first one investigating the different substrate characteristics of trigger sites in idiopathic RVOT VT and ARVC by the NCM method. This study has two main findings. First, the conduction time and distance from EA to BO site was longer in the ARVC cases. Second, the negative deflection of local VUE at the EA site was steeper in the RVOT VT patients. These results indicated potential subendocardial origin of the triggers in patients with ARVC.

### New Insights of VT Triggers From NCM

#### Different trigger characteristics from noncontact mapping

Idiopathic RVOT tachycardia is a nonfamilial and benign condition whereas ARVC is an inherited cardiomyopathy characterized by malignant ventricular arrhythmias and sudden death. Therefore, the differentiation between these two diseases, especially at the early stage, is extremely important. Previous studies have demonstrated the difference in electrophysiology characteristics between RVOT tachycardia and ARVC patient [17–21]. Corra et al. has used electroanatomical voltage mapping to identify RVOT tachycardia due to concealed ARVC by detecting RVOT electroanatomical scars that correlate with fibrofatty myocardial replacement at endomyocardial biopsy [21]. More recently, Zhang et al has demonstrated NCM-guided RVOT tachycardia ablation is highly effective and clinical success is best achieved by ablating the breakout site [8]. In their study, the mean distance between EA and BO were greater in patients who had recurrent ventricular arrhythmia at 12 months compared to patients who were free from recurrent arrhythmia. Another study conducted by Okumura et al [9] has also showed that the quantitative and qualitative analysis of the local virtual unipolar Eg from NCM may help to differentiate between RVOT and non-RVOT origin of VT as well as to predict both successful and potentially difficult ablation sites from the RVOT. In our study, the mean distance betwwen EA and BO sites in RVOT group was 7.6±4 mm, which is similar to the one observed in study of Zhang et al (8.4±5.7mm) [8]. The longer distance and conduction time from EA to BO sites in the ARVC group could be explained by the characteristic subtrate in ARVC. Both Corrado et al. [22] and Tandri et al. [23] have demonstrated fractionation of Eg and conduction delay in ARVC, respectively. Delayed RV activation is demonstrable in ARVC subjects even in the absence of global functional abnormalities and RV endocardial scar [23]. Therefore, a long distance and delayed conduction time from EA to BO sites could serve as a marker for differentiation between early ARVC and idiopathic RVOT VT.

In addition, histopathologic studies have demonstrated an epicardial predominance of the fibrofatty tissue in ARVC, suggesting that the disease process typically begins in the epicardium and progresses toward the endocardium [24–27]. Several studies have reported that combination of endo- and epicardial ablation results in better efficacy of ablation [28–29]. An animal study was conducted to determine if the characteristics of reconstructed unipolar electrograms from the noncontact mapping system can be used to detect epicardial electrical activation in a canine heart [30]. The authors concluded that the slurred activation and less slope of the virtual unipolar electrogram may indicate the epicardial in origin. Okumura et al [9] have shown that a deep origin of PVC/VT resulted in smaller VUE slope at the EA site. In the present study, in the ARVC endocardial RVOT, the smaller EA Unipolar Eg slope (manifested by a gentle negative deflection) suggested that the triggers of ARVC may originate from mid- or subepicardial myocardium. This hypothesis was supported by the fact that more radiofrequency applications were required to render VT non-inducible in ARVC patients and 3 of them needed an epicardial ablation.

#### Global substrate mapping

In the present study, we used the peak negative voltage as a recording technique for the voltage mapping of the RV substrate [31]. Our previous studies have demonstrated that individual normalization with a maximal PNV improved the accuracy of predicting the slow conduction region compared with the fixed value of the PNV [10,11,31]. This is compatible with the previous study of myocardial infarction model [32,33]. Relative ratio was used as a LVZ criteria to account for functional variation in unipolar peak negative voltage and LVZ of both beat to beat and patient to patient. A ratiometric PNV of 37% of the maximal PNV was found to best predict slow conduction [11]. Recently, Sivagangabalan G et al [34]. demonstrated that dynamic substrate mapping using the NCM, for sites within 40mm of the array, is comparable to the CARTO contact system in differentiating normal myocardium and scar in a chronic ovine model. A recent study of our group has demonstrated that endocardial unipolar PNV (LVZ) could predicts abnormal epicardial substrates in ARVC patients [35]. In our present study, the focal triggers in ARVC patients were found to originate from the LVZ; this finding support our hypothesis that these triggers came from a subepicardial or transmural diseased substrate.

In our study, there was no difference in voltage map between ARVC group and idiopathic RVOT VT group. The diagnosis of ARVC group was based on the modified Task Force criteria for diagnosis of ARVC. Endocardial voltage data is difficult to differentiate these two diseases. The results were controversial in previous studies. Boulos et al. compared electroanatomic findings in patients with an ultimate diagnosis of idiopathic RVOT tachycardia with those in patient who had established ARVC/D. They found that mapping results were in concordance with diagnosis [36]. Corrado et al. found that an early/minor form of ARVC may present clinically as RVOT tachycardia in the absence of RV dysfunction, thus mimicking idiopathic RVOT tachycardia. 3D electroanatomic voltage mapping may identify low voltage area in this patient group [21]. In previous study, 35% of patients who fulfilled the Task Force diagnostic criteria for ARVC showed no evidence of electroanatomic low-voltage regions [22].

### Clinical implication

Our study has provided a diagnostic tool which may help to differentiate between idiopathic RVOT VT and ARVC, especially in the early stage. Based on the result of our study we would propose that an EA to BO distance of >6mm and EA slope_5_ of ≤2.2mV should raise the suspicion of ARVC. NCM demonstrated that, a focal trigger with a slowing, slurred QS unipolar electrogram and long distance from EA to BO suggested a sub-endocardial in origin, which corresponded to the difficulty in endocardial ablation. Epicardial ventricular arrhythmias may be manifested as endocardial breakout in some patients. In these patients, epicardial ablation procedure could be required to achieve elimination of triggers.

### Study limitations

Firstly, the diagnosis of idiopathic RVOT VT was mainly clinical and not confirmed by MRI or endomyocardial biopsy. The overlap between the idiopathic RVOT VT and early stage ARVC may confound the result of our analysis. Secondly, the lower RV function may affect the arrhythmogenic substrate in ARVC group. Finally, our novel parameter differentiating between ARVC and idiopathic RVOT VT needs to be validated in larger studies.

### Sources of funding

This work was supported by Ministry of Science and Technology of Taiwan support for the Center for Dynamical Biomarkers and Translational Medicine, National Central University, National Yang-Ming University, and Taipei Veterans General Hospital (MOST 103-2911-I-008-001, 103-2314-B-010-048-MY3, 102-2314-B-010-056-MY2); Grant of Taipei Veterans General Hospital (V103E7-003); Joint foundation of Taipei Veterans General Hospital and National Taiwan University Hospital (VN103-04).

## Conclusions

The result of our study supports the conclusions of previous studies that ARVC and RVOT tachycardia are fundamentally different entities that can usually be distinguished. The substrate characteristics of PVC trigger may help to differentiate between idiopathic RVOT VT and ARVC. The slowing and slurred QS unipolar Eg and longer distance from EA to BO in RVOT endocardium suggest that the triggers of ARVC may originate from mid- or sub-epicardial myocardium. More extensive ablation to the trigger site was required in order to create deeper lesions for a successful outcome in ARVC group.
